# The impact of the combination of income and education on the incidence of coronary heart disease in the prospective Reasons for Geographic and Racial Differences in Stroke (REGARDS) cohort study

**DOI:** 10.1186/s12889-015-2630-4

**Published:** 2015-12-29

**Authors:** Marquita W. Lewis, Yulia Khodneva, Nicole Redmond, Raegan W. Durant, Suzanne E. Judd, Larrell L. Wilkinson, Virginia J. Howard, Monika M. Safford

**Affiliations:** Department of Human Studies, School of Education, College of Arts and Sciences, University of Alabama at Birmingham, 901 13th Street South, Birmingham, AL 35294-1250 USA; Department of Medicine, School of Medicine, University of Alabama at Birmingham, 1720 2nd Ave South, Birmingham, AL 35294-4410 USA; Department of Biostatistics, School of Public Health, University of Alabama at Birmingham, 1665 University Boulevard, Birmingham, AL 35294-0022 USA; Department of Epidemiology, School of Public Health, University of Alabama at Birmingham, 1665 University Boulevard, Birmingham, AL 35294-0022 USA

**Keywords:** Coronary heart disease, Myocardial infarction, Risk factors, Income, Education, Social determinants of health, Health disparities

## Abstract

**Background:**

We investigated the association between income-education groups and incident coronary heart disease (CHD) in a national prospective cohort study.

**Methods:**

The REasons for Geographic And Racial Differences in Stroke study recruited 30,239 black and white community-dwelling adults between 2003 and 2007 and collected participant-reported and in-home physiologic variables at baseline, with expert adjudicated CHD endpoints during follow-up. Mutually exclusive income-education groups were: low income (annual household income <$35,000)/low education (< high school), low income/high education, high income/low education, and high income/high education. Cox models estimated hazard ratios (HR) for incident CHD for each exposure group, examining differences by age group.

**Results:**

At baseline, 24,461 participants free of CHD experienced 809 incident CHD events through December 31, 2011 (median follow-up 6.0 years; interquartile range 4.5–7.3 years). Those with low income/low education had the highest incidence of CHD (10.1 [95 % CI 8.4–12.1]/1000 person-years). After full adjustment, those with low income/low education had higher risk of incident CHD (HR 1.42 [95 % CI: 1.14–1.76]) than those with high income/high education, but findings varied by age. Among those aged <65 years, compared with those reporting high income/high education, risk of incident CHD was significantly higher for those reporting low income/low education and low income/high education (adjusted HR 2.07 [95 % CI 1.42–3.01] and 1.69 [95 % CI 1.30–2.20], respectively). Those aged ≥65 years, risk of incident CHD was similar across income-education groups after full adjustment.

**Conclusion:**

For younger individuals, low income, regardless of education, was associated with higher risk of CHD, but not observed for ≥65 years. Findings suggest that for younger participants, education attainment may not overcome the disadvantage conferred by low income in terms of CHD risk, whereas among those ≥65 years, the independent effects of income and education are less pronounced.

## Background

Coronary heart disease (CHD) is the most common type of cardiovascular disease globally, and a leading cause of death for adults in the United States, accounting for 1 in every 6 deaths in 2010 [1–3]. Each year, an estimated 620,000 individuals will have their first hospitalized myocardial infarction (MI) or CHD death and another estimated 150,000 individuals will have their first silent MI [3]. Since the 1970’s, the U.S. has seen substantial declines in CHD mortality, but these rates of decline have varied, and in some cases increased [4–7], by race, sex, socioeconomic status (SES), and region [3, 8–11]. Numerous studies have found socioeconomic differences in the rates of incident CHD events [8, 12–14].

One potential promoter of these disparities is SES. Low income and low education have both been associated with higher CHD risk. People with low income are more likely to have CHD risk factors such as cigarette smoking, diabetes, high blood pressure, hyperlipidemia, suboptimal diets, inactivity, depressive symptoms, and stress [15, 16]. Participants in the Atherosclerosis Risk in Communities study with low income had a higher risk of non-fatal MI and cardiac death [14]. In the REasons for Geographic And Racial Differences in Stroke (REGARDS) study, participants with annual income <$35,000 had a higher incidence of CHD and CHD mortality compared to participants with annual income ≥$35, 000 (CHD incidence of 7.9 vs. 5.1 per 1000 person-years and mortality rate of 22.5 vs. 8.7 per 1000 person-years, respectively) [17]. Individuals who never completed high school are less likely to have health insurance. Furthermore, they are more likely to have lower levels of literacy, a decreased understanding of diseases and heart attack symptoms, and more physician recommendations [8, 18, 19]. Previous studies have reported significantly higher CHD risk for those with less than a high school education compared with high school graduates [20]. When studied together, income and education are complementary indicators used to define the multidimensional constructs of SES. Investigators have suggested that the observed health effects of income and education may be proxies for differences in employment, housing, access to nutritious food, and health insurance. Previous findings have suggested that individuals possessing multiple adverse social determinants such as low income and low education may be at increased risk for poor health outcomes [21, 22].

Despite the well-described risks of CHD associated with low income and low educational attainment, few studies have examined the combined effects of both low income and low education. Specifically, it is unclear whether excess risks associated with low income and/or education are attributable to higher risk factor burden, or whether additional unmeasured deleterious influences play a role. Further, it is unclear whether these relationships differ by age, gender, or race. Therefore, this study investigated the association between the combined effects of income and education and CHD events among black and white participants from the REGARDS study. We hypothesized that the combination of low income plus low education is more strongly associated with the incidence and risk of CHD than either low education or low income alone, or neither, after adjusting for known CHD risk factors. We also hypothesized that these relationships would differ for those <65 and ≥65 years of age, reflecting working age versus retired persons, for whom the meaning of annual household income differs.

## Methods

### Study sample

The REGARDS study is a prospective cohort study designed to study factors contributing to the geographic and racial differences in stroke incidence and mortality; the REGARDS- MI ancillary study examines CHD outcomes. Details are described elsewhere [9, 23, 24]. Briefly, the aims of the REGARDS-MI study are to estimate region and race-specific rates of definite or probable MI and CHD death, and to identify potential explanatory factors for racial differences in CHD. The cohort includes 30,239 community dwellers age ≥45 years residing in the 48 contiguous US states, with oversampling of the southeastern Stroke Belt (North Carolina, South Carolina, Georgia, Alabama, Mississippi, Tennessee, Arkansas, and Louisiana) [25]. Recruitment was conducted between 2003 and 2007 and oversampled blacks. The resulting sample was 42 % black (*n* = 12,490) and 55 % women (*n* = 16,612). Baseline data collection was completed using computer-assisted telephone interviews (CATI) which assessed demographic information, medical history, and health status. Following the CATI, trained health care professionals conducted an in-home examination using standardized, quality-controlled protocols to collect physiologic measures (blood pressure, height, and weight), blood and urine samples, electrocardiograms, and medication use by pill bottle review. All blood and urine samples were centrally analyzed at the University of Vermont. Electrocardiograms were centrally analyzed at Wake Forest University. Standardized follow-up telephone contacts were conducted every 6 months to detect hospitalizations and deaths. The study protocol was reviewed and obtained ethical approval by the University of Alabama at Birmingham Institutional Review Board and all participating institutional review boards, and all participants provided written informed consent. Additional information on the REGARDS study is available at www.regardsstudy.org.

This study was restricted to those free of CHD at baseline, defined as self-report of a previous MI or coronary revascularization procedure, or evidence of MI on the baseline ECG.

### Outcome

The outcome for this analysis was incident acute CHD, defined as definite or probable MI or acute CHD death. Briefly, during the follow-up contacts, any report of a heart-related event, death, hospitalization or emergency department visit prompted retrieval of medical records. Information for determination of CHD death included medical history, death certificates, autopsy reports, interviews with family members or proxies, and the National Death Index [26]. Two adjudicators assessed all cases and disagreements were adjudicated by committee. Adjudication of events was conducted by a team of experts using methods previously described [9, 26, 27]. REGARDS-MI follows an American Heart Association consensus statement that provides definitions of these endpoints for epidemiologic and clinical research studies [26]. Briefly, MI is determined by considering clinical signs and symptoms consistent with ischemia; ECG findings guided by the Minnesota code; and a rising and/or falling pattern of biomarkers, most often troponin, with a peak at least twice the lowest upper limit of normal over at least 6 hours (diagnostic enzymes). Definite MIs were those with diagnostic cardiac enzymes or ECG. Probable MIs were those with elevated but not diagnostic (i.e., equivocal) enzymes with a positive but not diagnostic electrocardiogram; or, if enzymes were missing, with a positive ECG in the presence of ischemic signs or symptoms. Only definite or probable MIs were included as events in this study. Adjudicators maintained a kappa statistic reflecting agreement of at least 0.8.

### Primary exposure

The primary exposure was the combination of baseline income and education. Low income was defined as annual household income <$35,000, a threshold established to be meaningful for CHD outcomes in a prior REGARDS study [17], and low education was defined as less than a high school education. Previous studies have reported significantly higher CHD risk for those with less than a high school education compared with high school graduates [28]. These two variables were combined into four exposure groups: 1) high education and high income, 2) high education and low income, 3) low education and high income, and 4) low education and low income.

### Covariates

Demographics included age, sex, race (black or white), region of residence (Nonbelt, Stroke Belt, Stroke Buckle), and urban residence [25]. Physiologic risk factors included systolic blood pressure, which was obtained after sitting for 5 min with both feet on the floor using an aneroid sphygmomanometer [24]; and body mass index (BMI), which was calculated as weight (kg) divided by the square of height (m^2^). Other physiologic measures included total cholesterol, high density lipoprotein [HDL] cholesterol, high sensitivity C-Reactive Protein (hsCRP, interassay CVs of 2.1 %–5.7 %) and urinary albumin to creatinine ratio (ACR, log-transformed and modeled as a continuous variable). Health behaviors included cigarette smoking (never, past, current), physical activity (none vs. any physical activity enough to work up a sweat in the past 7 days), and alcohol use (none, moderate [1–2 drinks/day for men, 1 drink/day for women], and heavy [>2 drinks/day for men and >1 drink/day for women). Medical history included diabetes (fasting glucose ≥126 mg/dl, nonfasting glucose ≥200 mg/dl, or treatment with a diabetes medication), vascular disease (self-reported history of stroke, peripheral arterial disease, aortic aneurism), antihypertensive medication use, insulin use, and statin use. Psychosocial measures included baseline depressive symptoms (measured with the 4-item Center for Epidemiologic Studies Depression scale with a score of 4 or greater indicating the presence of depressive symptoms); and perceived stress (measured with a 4-item version of the Cohen Perceived Stress Scale with a score >5 indicating high stress) [29]. Health insurance was classified as present or absent.

### Statistical analysis

Follow-up for this analysis was through December 31, 2011, and follow-up time for each participant was calculated from the date of the in-home visit to the date of an incident CHD event, death, or last follow-up contact. CHD incidence rates were calculated as the number of incident events divided by person-years at risk within each age-race-sex-region stratum, with 95 % confidence limits calculated assuming a Poisson distribution. Adjusted rates were standardized to the 2000 U.S. population. Baseline characteristics across the income-education groups were compared using the Chi-square test for categorical variables and analysis of variance for continuous variables. Incrementally adjusted Cox proportional hazards models estimated hazard ratios (HRs) for the income-education groups for incident acute CHD events. Initial unadjusted models included income-education groups only. Models were then constructed incrementally. Model 1 adjusted for geodemographics (sex, age, race, and geographic region of residence, urban residence). Model 2 added health behaviors (smoking, alcohol consumption, and physical activity) to the model 1 covariates. Model 3 added physiologic variables (BMI, systolic blood pressure, total and HDL cholesterol, log-transformed hsCRP, log-transformed ACR); medical history and treatment variables (vascular disease, diabetes, use of statins, use of antihypertensive medications, use of insulin); and psychosocial and health care access variables (depressive symptoms, perceived stress, insurance status) to the model 2 covariates. Approximately 11 % of the participants (*n* = 3,027) declined to provide income data; therefore, we used multiple imputation by chained equations with 10 datasets to estimate missing data [30, 31]. The assumptions of proportionality in the Cox models were tested and found not to be violated. Interactions were tested for income-education group and age, gender, and sex, Statistical significance for all analyses was set at *p* <0.05. Analyses were conducted using SAS version 9.3 (SAS Institute, Inc.) and Stata version 12 (StataCorp).

## Results

### Sample characteristics

Excluding the 5778 individuals with CHD at baseline resulted in an analytic sample of 24,461 participants. Baseline characteristics of the study sample overall and by income-education category are shown in Table 1. Only 293 (1.4 %) participants reported high income and low education. Compared with participants in other income-education categories, participants who reported both low income and low education were more likely to be black, have comorbidities (high blood pressure, diabetes, cardiovascular disease), take antihypertensive medication, use insulin, currently smoke, be physically inactive, and perceive more stress. Participants with lower income were more likely to be women, have more depressive symptoms, and be less likely to have health insurance.

**Table 1:** Baseline characteristics of REGARDS participants without coronary heart disease (*N* = 24,461), by Income-Education Category

Characteristics	Overall	High income-high education *n* = 11155	Low income^a^-high education *n* = 7955	High income-low education^b^ *n* = 293	Low income^a^- low education^b^ *n* = 2009	*P*-Value
*Demographics*						
Age, mean ± SD	64.1 ± 9.3	61.8 ± 8.7	65.7 ± 9.4	63.7 ± 9.5	67.9 ± 9.7	<.0001
Women, n (%)	12194 (57.0)	5469 (49.0)	5338 (67.1)	113 (38.6)	1274 (63.4)	<.0001
Black, n (%)	9054 (42.3)	3549 (31.8)	3947 (49.6)	128 (43.7)	1430 (71.2)	<.0001
Region of residence, n (%)						<.0001
Nonbelt	9563 (44.7)	5222 (46.8)	3472 (43.7)	127 (43.3)	742 (36.9)	
Stroke Belt^c^	7409 (34.6)	3594 (32.2)	2912 (36.6)	92 (31.4)	811 (40.4)	
Stroke Buckle^d^	4439 (20.7)	2338 (21.0)	1571 (19.8)	74 (25.3)	456 (22.7)	
Urban residence, n (%)	15213 (78.6)	7826 (77.4)	5760 (80.3)	202 (75.7)	1425 (79.3)	<.0001
*Physiologic risk factors*						
Systolic blood pressure, mm Hg, mean ± SD	127.1 ± 16.5	124.7 ± 15.3	129.0 ± 17.0	128.7 ± 16.1	132.2 ± 18.2	<.0001
Body mass index (kg/m^2^), mean ± SD	29.3 ± 6.2	28.9 ± 5.7	29.7 ± 6.7	29.5 ± 5.7	30.4 ± 6.8	<.0001
Total cholesterol (mg/dL), mean ± SD	195.1 ± 39.3	194.0 ± 37.3	196.7 ± 41.3	190.5 ± 39.9	193.1 ± 40.6	0.75
High density lipoprotein cholesterol (mg/dL), mean ± SD	52.8 ± 16.3	52.3 ± 16.3	53.1 ± 16.1	48.3 ± 14.6	51.6 ± 15.4	0.18
High sensitivity C-reactive protein, median [interquartile range]	2.2 [0.9–5.0]	1.8[0.8–4.2]	2.6[1.1–6.0]	2.6[1.3–5.7]	3.2[1.3–6.7]	<.0001
Urinary albumin to creatinine Ratio, median [interquartile range]	7.1 [4.5–14.5]	6.25[4.2–11.7]	7.9 [4.9–17.17]	6.9 [4.4–13.9]	9.3 [5.2–24.3	<.0001
*Health behaviors*						
Smoking status, n (%)						<.0001
Never	10036 (47.0)	5446 (49.0)	3676 (46.4)	99 (33.9)	815 (40.7)	
Past	8226 (38.5)	4456 (40.1)	2872 (36.2)	139 (47.6)	759 (37.9)	
Current	3085 (14.5)	1219 (11.0)	1382 (17.4)	54 (18.5)	430 (21.5)	
No Exercise, n (%)	6988 (33.1)	3097 (28.0)	2939 (37.5)	97 (34.0)	855 (43.4)	<.0001
Alcohol use, n (%)						<.0001
Heavy	887 (4.2)	576 (5.2)	255 (3.3)	10 (3.5)	46 (2.4)	
Moderate	7271 (34.6)	4784 (43.5)	2057 (26.4)	93 (32.8)	337 (17.2)	
None	12870 (61.2)	5628 (51.2)	5486 (70.4)	181 (63.7)	1575 (80.4)	
*Medical history*						
Diabetes, n (%)	4004 (19.4)	1548 (14.3)	1763 (23.1)	62 (21.8)	631 (32.9)	<.0001
Vascular disease, n (%)	1373 (13.7)	435 (3.90)	655 (8.23)	24 (8.19)	259 (12.9)	<.0001
Antihypertensive medication use, n (%)	10312 (48.6)	4680 (42.3)	4239 (53.9)	137 (47.6)	1256 (63.4)	<.0001
Insulin use, n (%)	953 (4.4)	316 (2.8)	445 (5.6)	11 (3.8)	181 (9.0)	<.0001
Statin use, n (%)	5980 (28.2)	3089 (27.9)	2180 (27.6)	86 (29.4)	625 (31.5)	0.0057
*Psychosocial measures and insurance coverage*						
Depressive Symptoms (CES-D ≥4), n (%)	2163 (10.2)	631 (5.7)	1074 (13.6)	26 (8.9)	432 (21.6)	<.0001
Perceived Stress, median score [interquartile range]	3.0[0–5.0]	2.0[0–4.0]	3.0[1.0–6.0]	3.0[0–5.0]	4.0[1.0–7.0]	<.0001
Insured, n (%)	1990 (93.0)	10840 (97.2)	7026 (88.4)	277 (94.5)	1764 (87.9)	<.0001

^a^Low income defined as annual household income <$35,000

^b^Low education defined <high school education

^c^Defined as the states of Alabama, Arkansas, Louisiana, Mississippi, Tennessee and the noncoastal regions within the states of North Carolina, South Carolina, and Georgia

^d^ Defined as the coastal regions within the states of North Carolina, South Carolina, and Georgia

*CI* confidence interval, *SD* standard deviation, *CES-D* center for epidemiologic studies depression

### CHD incidence rates

There were a total of 809 incident CHD events through December 31, 2011, with a median follow-up of 6.0 years (25th, 75th percentiles 4.5–7.3). The age-adjusted incidence rates of acute CHD per 1000 person-years for those with low vs. high income were 7.0 [95 % CI 6.3–7.8] and 5.3 [95 % CI 4.7–5.8], respectively, and for those with low vs. high education were 9.7 [95 % CI 8.3–11.3] and 5.6 [95 % CI 5.2–6.0], respectively. The age-adjusted incidence rates of acute CHD per 1000 person-years for each of the income-education exposure categories are shown in Table 2. Those with both low income and low education had the highest age-adjusted CHD incidence rate (10.1 [95 % CI: 8.4–12.1] per 1000 person-years), followed by those with high income and low education (8.1 [95 % CI: 4.8–13.7]), low income and high education (6.3 [95 %, CI: 5.6–7.1]) and high income and high education (5.12 [95 % CI: 4.7–5.8]).

**Table 2:** Incident CHD events, incidence rates, and hazard ratios for income-education categories e in REGARDS participants

	High income-high education *n* = 11155	Low income^a^-high education *n* = 7955	High income-low education^b^ *n* = 293	Low income^a^- low education^b^ *n* = 2009
Events, n	335	325	14	135
Age-adjusted Incidence Rate per 1000 person-years (95 % CI)	5.2 (4.7–5.8)	6.3 (5.6–7.1)	8.1 (4.8–13.7)	10.1 (8.4–12.1)
p-value for difference compared to high income/high education	*ref*	0.02	0.10	<.001
Models				
Crude Analysis (95 % CI)	*ref*	1.42 (1.22–1.65)	1.66 (0.96–2.86)	2.51 (2.08–3.03)
Model 1 HR (95 % CI)	*ref*	1.40 (1.19–1.64)	1.43 (0.84–2.45)	2.15 (1.75–2.63)
Model 2 HR (95 % CI)	*ref*	1.10 (0.93–1.29)	1.18 (0.69–2.02)	1.45 (1.18–1.80)
Model 3 HR (95 % CI)	*ref*	1.07 (0.92–1.27)	1.18 (0.69–2.03)	1.42 (1.14–1.76)

^a^Low income defined as annual household income <$35,000

^b^Low education defined as <high school education

Model 1 adjusts for sex, age, race, and geographic region of residence

Model 2 adjusts for smoking, physical activity, and alcohol consumption

Model 3 adjusts for body mass index, systolic blood pressure, total cholesterol, high-density lipoprotein cholesterol, C-reactive protein, albumin-to-creatinine ratio, baseline cardiovascular disease (stroke,, peripheral vascular disease, aortic aneurism), diabetes, statins, antihypertensive medications, insulin, insurance status, baseline depressive symptoms and perceived stress

*HR* hazard ratio, *CI* confidence interval

### Risk for incident CHD

In a crude model, the HR for incident CHD for individuals with low vs. high income was 1.61 (95 % CI: 1.41–1.85), and that for individuals with low vs. high education was 2.05 (95 % CI: 1.74–2.41). In a fully adjusted model including both covariates, the HR for low vs. high income was 1.08 (95 % CI: 0.92–1.27) and for low vs. high education was 1.30 (95 % CI: 1.09–1.56).

Risks for incident CHD for individuals in the four income-education categories are compared in Table 2. In the crude analysis, compared with those reporting both high income and high education, the HR for incident CHD among participants reporting low income and low education was 2.51 (95 % CI: 2.08–3.03), for high income and low education was 1.66 (95 % CI: 0.96–2.86), and for low income and high education was 1.42 (95 % CI: 1.22–1.65). Adjusting for demographic characteristics in model 1 (Table 2) had little effect on CHD risk among those with low income and high education, but risks demonstrated greater attenuation for those with high income and low education as well as those with both low income and low education. A larger attenuation was observed on addition of health behaviors (model 2). Further addition of physiologic measures, comorbidities, medication use, and insurance status (model 3) had little effect on the size of the HR. Only those with both low income and low education had significantly higher risk in the fully adjusted model (HR 1.42 [95 % CI: 1.14–1.76]). Interactions between age and income-education groups were significant (*p* <0.001), but not for race*income-education (*p* = 0.1827) and gender*income-education (*p* = 0.1096). The analysis was stratified at age 65 years, based on the age of retirement.

### Differences by age

The results of the analysis stratified on age 65 years are shown in Table 3. All characteristics differed across income-education categories except for statin use for those ≥65 years (*p* = 0.177). The incidence of CHD across the income-education categories differed by age group. For participants <65 years of age, the incidence of CHD was highest in those reporting both low income and low education (10.8 [95 % CI: 8.0–14.6] per 1000 person-years), but for those ≥65 years of age, the incidence of CHD was highest in those reporting high income and low education (15.8 [95 % CI: 8.8–28.6] per 1000 person-years). Among those age <65 years, the lowest incidence rate was observed among those with both high income and education (3.0[95 % CI: 2.6–3.6] per 1000 person-years) and with high income and low education (3.0 [95 % CI: 1.0–9.4] per 1000 person-years), but among those age ≥65, the lowest incidence was observed among those with low income and high education (7.4 [6.4–8.5] per 1000 person-years).

**Table 3:** Characteristics of income-education groups and association with incidence of CHD stratified by 65 years

	High income-high education	Low income^a^-high education	High income-low education^b^	Low income^a^-low education^b^	*P*-Value
Age			<65		
N	7234	3696	167	723	
Age, mean ± SD	56.6 ± 5.00	57.5 ± 4.94	56.9 ± 5.14	58.4 ± 4.70	<0.001
Black, n (%)	2468 (34.1)	2115 (57.2)	69 (41.3)	525 (72.6)	<0.001
Women, n (%)	3844 (53.1)	2516 (68.1)	78 (46.7)	494 (68.3)	<0.001
Had diabetes, n (%)	932 (13.3)	878 (24.7)	28 (17.1)	220 (31.7)	<0.001
Hypertension, n (%)	3157 (43.7)	2161 (58.6)	79 (47.3)	512 (71.0)	<0.001
Antihypertensive medication use, n (%)	2676 (37.3)	1845 (36.7)	61 (37.4)	445 (62.4)	<0.001
Statin use, n (%)	1758 (24.5)	881 (24.0)	39 (23.4)	199 (27.9)	0.177
Current Smoking, n (%)	971 (13.5)	904 (34.5)	41 (24.7)	231 (32.0)	<0.001
# of Events	131	137	3	44	
Person-years	42525.61	20566.18	972.03	3803.07	
Incidence Rate per 1000 person-years (95 % CI)	3.0 (2.6–3.6)	6.4 (5.4–7.6)	3.0 (1.0–9.4)	10.8 (8.0–14.6)	
Crude HR (95 % CI)	*ref*	2.23 (1.76–2.83)	0.96 (0.31–3.02)	3.58 (2.56–4.99)	
Fully adjusted HR (95 % CI)^c^	*ref*	1.69 (1.30–2.20)	0.69 (0.22–2.16)	2.07 (1.42–3.01)	
Age			≥65		
N	3921	4259	126	1286	<0.001
Age, mean ± SD	71.4 ± 5.35	72.8 ± 5.87	72.7 ± 5.76	73.3 ± 6.12	<0.001
Black, n (%)	1082 (27.6)	1832 (43.0)	59 (46.8)	905 (70.4)	<0.001
Women, n (%)	1625 (41.4)	2822 (66.3)	35 (27.8)	780 (60.7)	<0.001
Had diabetes, n (%)	616 (16.2)	885 (21.7)	34 (28.1)	411 (33.6)	<0.001
Hypertension, n (%)	2321 (59.4)	2782 (65.5)	87 (69.1)	934 (72.7)	<0.001
Antihypertensive medication use, n (%)	2005 (51.4)	2394 (56.8)	76 (60.1)	811 (64.0)	<0.001
Statin use, n (%)	1332 (34.3)	1299 (30.7)	47 (37.3)	426 (33.5)	0.0031
Current Smoking, n (%)	248 (6.34)	478 (11.3)	13 (10.3)	199 (15.5)	<0.001
# of Events	204	188	11	91	
Person-years	23226.34	24270.08	668.03	6874.68	
Incidence Rate (95 % CI)	9.0 (7.8–10.3)	7.4 (6.4–8.5)	15.8 (8.8–28.6)	12.3 (10.0–15.1)	
Crude HR (95 % CI)	*ref*	0.88 (0.73–1.07)	1.82 (1.02–3.25)	1.58 (1.27–1.98)	
Fully adjusted HR (95 % CI)^c^	*ref*	0.82 (0.67–1.01)	1.39 (0.77–2.49)	1.16 (0.90–1.50)	

^a^Low income defined as annual household income <$35,000. ^b^Low education defined <high school education

^c^Fully adjusted adjusts for sex, age, race, geographic region of residence, smoking, physical activity, alcohol consumption, body mass index, systolic blood pressure, total and high-density lipoprotein cholesterol, C-reactive protein, albumin-to-creatinine ratio, baseline cardiovascular disease (stroke, periphery artery disease, aortic aneurism), and diabetes, insurance status, statins, antihypertensive medications, insulin use, baseline depressive symptoms and perceived stress

*CI* confidence interval, *SD* standard deviation

Among the younger age group, compared to those reporting both high income and education, the hazards were statistically significantly higher for those with low income, regardless of education, in both the crude and fully adjusted analyses (Table 3). In contrast, for the older age group, only those with low education, regardless of income, had an increased risk of incident CHD in the crude analysis, but these risks were attenuated with full adjustment.]

## Discussion

In this study, participants reporting both low income and low education had nearly twice the incidence of CHD as participants reporting both high income and high education. The differences in incidence for either low or high income and education alone were not as pronounced as those observed when considering the combined effects of education and income. These results suggest the importance of examining income and education together, as those with low attainment in both categories were observed to have the highest risk. Notably, these effects varied by age, with low income and low education associated with the greatest CHD risk for participants <65 years, and high income and low education associated with the greatest CHD risk for participants ≥65 years. Additionally, among those <65 years, participants with both low income and education had over a threefold greater incidence of CHD compared to those with both high income and education. Multivariable models adjusting for a host of potential confounders demonstrated that those with both low income and education still had twice the risk of incident CHD compared to those with high income and education. These differences were not as pronounced among those ≥65 years. Our findings suggest that among younger people, education attainment may not overcome the disadvantage conferred by low income in terms of CHD risk, whereas among older people, the independent effects of income and education are less pronounced, especially after accounting for confounders.

Few studies have examined the combined effect of education and income on incident CHD across different age groups in a national cohort. Consistent with our findings, income and education were inversely associated with CHD risk in cohorts from the U.S. and Finland [14]. A German cohort examined the association of education level with long-term mortality after the first MI in adults aged 28–74 years. Participants with 13 or more years of school had significantly longer survival and the association between MI mortality and low education for younger participants was nonsignificant [13]. In contrast, in a cohort from Minnesota, low income and low education were independently associated with increased CHD mortality, but the effect of education was not significant in the fully adjusted model [22]. Although prior research suggested that income and education have significant effects on CHD risk, our study has further elucidated the combined impact of income and education.

Few studies have examined age-related differences in the associations of both income and education with incident CHD. We found that low income and low education were associated with the highest CHD risk at younger ages, < 65 years, but not at older ages. Proposed explanations for the association of the increased CHD risk and low education observed in older adults include greater illness burden [22], less knowledge of CHD signs and symptoms [13, 19, 32], and low health literacy [33]. Other studies have shown a “selective survival” or a “mortality crossover” effect, as evidenced by longer life expectancy among the oldest old adults (≥75 years) regardless of income because the surviving elderly are healthier than their younger counterparts [34, 35]. Additionally, adults ≥65 years are more likely to be retired and to therefore have a fixed income comprised of employer provided benefits, social security, and personal savings, and their expenses may be lower than those of younger individuals with families. This makes annual household income less reliable as an indicator of wealth in this age group. Additionally, expenditures associated with healthcare are often completely or partially covered by Medicare benefits, military benefits, or private insurance. In 2015, more than 50 million Americans had health coverage through Medicare, and the majority of these beneficiaries were 65 years and older [36]. Middle aged adults are more likely to have variable income and unpredictable expenditures related to caregiving for older as well as younger relatives, and many chronic diseases emerge in middle age, creating multiple sources of financial demand, which may explain the greater influence of income in the younger age group. In 2011, approximately 35 % of multigenerational US homes had a householder providing care for an aging parent or parent-in-law [37].

It is worth noting that comparing income and education effects across studies is challenging [22, 38]. In our cohort, education and income data were based on self-reported education and annual household income, respectively. The definition of SES varies across studies, ranging from income only [34]; employment status, household income, and educational level [39]; income and education [14]; neighborhood income; and census tract income and education data [21]. Similarly, measurements of SES are multidimensional and lack standardization across health research, especially across international studies [40, 41]. Additionally, we used binary income categories based on a recent REGARDS that indicated an effect modification at an annual household income above and below $35,000, and deleterious health effects of stress were observed below (but not above) this level [17]. In 2003 and 2007 the Federal poverty line for a household of four was $18,400 and $20,650, respectively. Our $35,000 annual household income level is 70–98 % in excess of the Federal Poverty Line for the corresponding years; similar thresholds may not be observed in other cohorts [38].

Our findings have implications for efforts to reduce disparities in health outcomes through targeted health programming and initiatives for risk groups that have both low income and education. Reducing CHD mortality and improving cardiovascular health is a national objective. The American Heart Association aims to reduce stroke and cardiovascular mortality by 20 % and improve cardiovascular health by 20 % for all Americans by 2020 through health behavior modification [42]. The American Heart Association 2020 goal articulates a need to strategically address populations at highest risk. Likewise, the Million Hearts initiative of the U.S. Department of Health and Human Services focuses on partnerships with communities, healthcare systems, community organizations, and federal agencies to prevent 1 million heart attacks and strokes by 2017 [43].

Some limitations should be considered in the interpretation of these findings. Income and education as well as several other variables were self-reported with known susceptibility to reporting bias. Secondly, the REGARDS study was not designed as a surveillance study, thus it is likely that some events were not detected, resulting in underestimation of true CHD incidence rates. We note that this study focused primarily on tests of association, which are less prone to biases because of incomplete event ascertainment. Last, although REGARDS participants are distributed nationally, cohort study participants may differ from the general population, affecting generalizability. We note that healthy volunteer effects would likely bias our findings toward the null, thus associations in the general population may be even greater than those reported here.

## Conclusion

Overall, we found that the combination of both low income and low education were associated with higher risk of incident CHD than either low income or low education alone. The effects of the combination of low income and low education were most pronounced in individuals under the age of 65 years. Our findings support the targeting of resources for individuals with both low income and low education, especially those less than 65 years of age.

### Ethics, consent and permissions

The study protocol was reviewed and obtained ethical approval by the University of Alabama at Birmingham Institutional Review Board and all participating institutional review boards, and all participants provided written informed consent. Additional information on the REGARDS study is available at www.regardsstudy.org.

## Abbreviations

95 % CL: 95 % confidence limits
ACR: albumin-to-creatinine ratio
BMI: Body Mass Index
CHD: Coronary heart disease
CRP: C-reactive protein
HR: Hazard ratio
MI: Myocardial Infarction
REGARDS: Reasons for Geographical and Racial Differences in Stroke
SD: Standard deviation
SES: Socioeconomic Status
